# Mutability of druggable kinases and pro-inflammatory cytokines by their proximity to telomeres and A+T content

**DOI:** 10.1371/journal.pone.0283470

**Published:** 2023-04-27

**Authors:** Ian McKnight, Regan Raines, Hunter White, Nasim Nosoudi, Chan Lee, Peter H. U. Lee, Joon W. Shim

**Affiliations:** 1 Department of Biomedical Engineering, College of Engineering and Computer Sciences, Marshall University, Huntington, West Virginia, United States of America; 2 Department of Anesthesia, Indiana University Health Arnett Hospital, Lafayette, Indiana, United States of America; 3 Department of Cardiothoracic Surgery, Southcoast Health, Fall River, Massachusetts, United States of America; 4 Department of Pathology and Laboratory Medicine, Brown University, Providence, Rhode Island, United States of America; Osmania University, Hyderabad, India, INDIA

## Abstract

Mutations of protein kinases and cytokines are common and can cause cancer and other diseases. However, our understanding of the mutability in these genes remains rudimentary. Therefore, given previously known factors which are associated with high mutation rates, we analyzed how many genes encoding druggable kinases match (i) proximity to telomeres or (ii) high A+T content. We extracted this genomic information using the National Institute of Health Genome Data Viewer. First, among 129 druggable human kinase genes studied, 106 genes satisfied either factors (i) or (ii), resulting in an 82% match. Moreover, a similar 85% match rate was found in 73 genes encoding pro-inflammatory cytokines of multisystem inflammatory syndrome in children. Based on these promising matching rates, we further compared these two factors utilizing 20 *de novo* mutations of mice exposed to space-like ionizing radiation, in order to determine if these seemingly random mutations were similarly predictable with this strategy. However, only 10 of these 20 murine genetic loci met (i) or (ii), leading to only a 50% match. When compared with the mechanisms of top-selling FDA approved drugs, this data suggests that matching rate analysis on druggable targets is feasible to systematically prioritize the relative mutability—and therefore therapeutic potential—of the novel candidates.

## Introduction

Protein kinases are enzymes which catalyze transfer of the ɣ-phosphate of adenosine triphosphate (ATP: energy carrying molecule) to amino acid side chains in substrate proteins such as serine, threonine, and tyrosine residues. Many critical protein kinase drug targets in cancer and non-cancerous conditions—including receptor kinases, enzymes, ion channels, and transporters—have been long identified. Thus, when the first kinase inhibitor, imatinib, acquired Food and Drug Administration (FDA) approval twenty years ago, the groundwork was laid for protein kinases to become major modern druggable targets [1]. In the case of imatinib and many other early kinase inhibitors, in the initial period of drug development, they achieved success in extending survival rates of patients with cancer by targeting the wild type (WT) sequences of their target kinase [1–6].

However, kinase mutations can potentially determine which patients benefit the most from certain drugs. For example, epidermal growth factor receptor (EGFR) inhibitors, such as gefitinib (market brand name, Iressa) and erlotinib (brand name, Tarceva)—which were both approved for the treatment of non-small cell lung cancer—are prime examples of this mutation-driven kinase inhibitor modification. These drugs were originally designed to inhibit the wild-type form of the EGFR (134 kDa), which was shown to be overexpressed in many tumors, leading to poor outcomes [2, 6]. However, the profound sensitivity of the tumors to these drugs was actually primarily associated with specific EGFR mutations that are found in 10–15% of non-Asian and 30–50% of Asian patients with lung cancers [3–5, 7]. This led to the targeted clinical use of these inhibitors in patients with these EGFR mutations, thereby achieving even better outcomes [1].

Mutations in EGFR or other kinases, which are crucial components in the activation of the classical mitogen-activated protein (MAP) kinase cascade, are prime examples of how soluble ligands with less mutability are associated with important downstream signaling pathways—and through which mutations can cause cancer. More specifically, the MAP kinase cascade is frequently hyperactivated in lung and other cancers owing to the overexpression or mutation of receptor tyrosine kinases, such as EGFR, anaplastic lymphoma kinase (ALK), MET proto-oncogene (MET), and other downstream effectors, which are most commonly rat sarcoma virus gene (RAS) and proto-oncogene B-Raf (BRAF). The drugs that target EGFR, ALK, and MET, including gefitinib, crizotinib, and capmatinib, respectively—as well as approved inhibitors for BRAF—all modulate the functions of the MAP kinase cascade [1]. Soluble ligands—and specifically cytokines—can potentiate mutations in their ligand binding sites [8] and affect the function of downstream molecules involved in the MAP kinase cascade as well, thereby generating similar negative outcomes [1]. However, cytokines also serve a prevalent function in the mediation of inflammation as well as response to both wild-type and mutated viral antigens—which has been demonstrated through recent cases of COVID-19 [9].

The B.1.617.2 (Delta) variant of COVID-19 has caused a serious problem in re-escalating the infection rate globally, which is due primarily to the high ‘transmission rate’ even post-vaccine administration [10]. When multisystem inflammatory syndrome in children (MIS-C) [9] first became an issue, the COVID-19 vaccines had not yet been released and adults comprised the majority of confirmed cases. This effect was thought to be due to differences in the innate immune response—the body’s crude but swift reaction to pathogens—between adults and children [11]. For now, there is no clear evidence that children are more vulnerable to or more affected by Delta in comparison with earlier variants. Like all viruses, SARS-CoV-2 is constantly mutating and becoming better at evading host defenses, and that makes understanding the additional protective benefits seen in children important to examine, particularly in regard to how mutation rates can impact disease outcome [11].

Greater lifespans in higher-order metazoans correspond with higher gradual accumulation of cellular damage, thereby leading to decreases in tissue function (cellular senescence) and fitness as an organism ages [12]. Like bacteria, somatic cells can adapt to environmental pressure by enhancing their mutability [12]. This adaptive amplification of erroneous change, which increases as a cell senesces, is due to a native inducible chromosomal instability mechanism, and is defined as adaptive mutability [13–18]. This concept of mutability has been applied to cancer research, thereby permitting the reclassification of adaptive mutability [19–21] into ‘relative mutability’ [22], as explored by our recent investigations [23, 24]. We defined relative mutability using two factors associated with high mutation rates in human chromosomes, enabling the analysis of both inherited and somatic mutations. Previously, several factors have been reported to be associated with high mutation rates in human genomes, including 1) recombination rate [25], 2) proximity to a telomere, and 3) high adenine/thymine (A+T) content [24, 26].

Among these factors, we have previously demonstrated that proximity to a telomere [27] and nucleotide composition (A+T content) can explain some of the genetic mutations linked to monogenic and/or polygenic diseases [23, 28]. Therefore, since both natural mutations due to cellular senescence and disease-driven mutations such as those in COVID-19 merit additional exploration, we aim to use this dual factor analysis to examine both protein kinases and pro-inflammatory cytokines. We also aim to analyze *de novo* mutations using this technique, in order to explore the efficacy of this technique in examining generational mutations due to environmental insults, such as radiation [29].

The National Institute of Health (NIH) of the United States has recently released more than 300 understudied druggable genomes entitled as the “Commercializing Understudied Proteins from the Illuminating the Druggable Genome” project (PA-19-034). From that list, 129 druggable candidates were classified as protein kinases. However, whether the aforementioned factors can prioritize which of these 129 protein kinases are the least mutable—in order to prioritize druggable targets for the purpose of future commercialization—as was seen previously with kinase inhibitors for EGFR mutations, remains to be elucidated. The same is true for the 73 genes encoding pro-inflammatory cytokines related to MIS-C. Thus, here, we investigate how many genes encoding the 129 druggable kinases and 73 pro-inflammatory cytokines studied match with one, both, or neither of the two predictive factors: (i) proximity to telomeres and (ii) high A+T content. This may help prioritize the druggable kinases based on the relative mutability predicted by these two factors, as well as potentially identify prominent cytokine mutability in MIS-C cases.

Next, to highlight the impact of radiation—such as that experienced during space travel—on genetic mutation, we compared this human genetic data with 20 *de novo* mutations in mice exposed to ionizing radiation [29]. Ultimately, we aim to show that the genomic characteristics of several top-selling drugs targeting protein kinases and those of commercialized drugs targeting soluble ligands or cytokines can be predicted and prioritized based on mutability. We envision that matching rate analysis of the druggable genome will not only be a useful tool to systematically prioritize the relative mutability of druggable protein kinases and cytokines, but that it will also permit the characterization of mutations due to genetic and/or environmental factors.

## Materials and methods

### Database, literatures, and open access software

The list of 129 candidate genes encoding protein kinases follows the classification and identification of 390 understudied druggable genomes in the publicly open NIH program announcement (PA-19-034). The literature survey was carried out with emphasis on multi-system inflammatory syndrome in children for pro-inflammatory cytokines [9] and ionizing radiation exposure in mice for *de novo* mutations evidently observed after environmental insult or radiation exposure mimicking space missions [29]. This literature survey was systematically conducted according to our previous methods [28].

For the measurements of the distance between the gene of interest and its telomere, as well as the calculation of A+T content, we utilized the NIH Genome Data Viewer (https://www.ncbi.nlm.nih.gov/genome/gdv/) and the GC content calculator (https://www.biologicscorp.com/tools/GCContent/#.XvctCi-z2uV), resulting in compositions of adenine and thymine along with the full-length sizes of the nucleotide [23].

### Approximation of proximity to a telomere

The biological basis for the apparently high mutation rate in human chromosomes has been previously described. We have followed the established method in approximating a gene’s proximity to a telomere [23]. As a result, in this study, we have calculated the nucleotide compositions of the gene encoding a protein kinase and focused on the position of the gene and its distal end locus of each arm (telomere) with the following premise [30]:

Ifrecombinationfrequencyislessthan(≤)50cM,genesarelinked: (1)

ifrecombinationfrequencyishigherthan50cM,genesarenotlinked, (2)

where 1 centimorgan (cM) ≅ 1 million base pair (Mbp) [31].

### Successive steps of workflow

Successive steps for the biophysical measurement of the distance to a telomere and the calculation of biochemical composition of the nucleotide followed the previous publications [22, 23, 28, 32]. Briefly, the list of target genes (transcript) is determined and tabulated in a spreadsheet with 10 columns—gene name, abbreviated gene identification (ID), chromosome, gene locus, telomere locus, gene to telomere (distance), Adenine, Thymine (%), A+T (%), full-length size (FL in bases). For each gene of interest, 1) The genome data viewer is used to obtain the access number such as ‘NM_ _ _ _’. Then, 2) the PubMed nucleotide database is opened and the previously obtained ‘NM_ _ _ _’ is entered to get the full-length nucleotide base sequences located at the bottom of the query result. The sequence is copied in its entirety to the clipboard and pasted at 3) the GC content calculator to derive A+T content (%). During the first step using the genome data viewer, the biophysical distance of a gene to its telomere is measured and entered into the spreadsheet—along with chromosome number, where the gene is located, as well as the full-length size of the molecule. In this fashion, the blanks in the table with 10 columns are successively filled out, forming a basis for data plot and statistical analyses.

### Data plot and statistical methods

Prism (version 9.5.1, GraphPad Software Inc.) was used to plot a bar graph and box and violin plot of the data obtained during analysis with the genome data viewer. Statistical analyses were performed using Prism as well. Normal distribution of the data was confirmed using Shapiro-Wilk normality test (α<0.05). A two-sided unpaired t-test was used for comparison of two different groups, unless stated differently. Tukey’s multiple comparisons test following one-way analysis of variance was used for comparison of more than two groups. The difference between data sets was considered significant at P<0.05; P values are identified in the figures and legends as *P<0.05, **P<0.01, ***P<0.005.

## Results

### 129 novel protein kinase genes satisfied two factors at 82%

**Fig 1:**
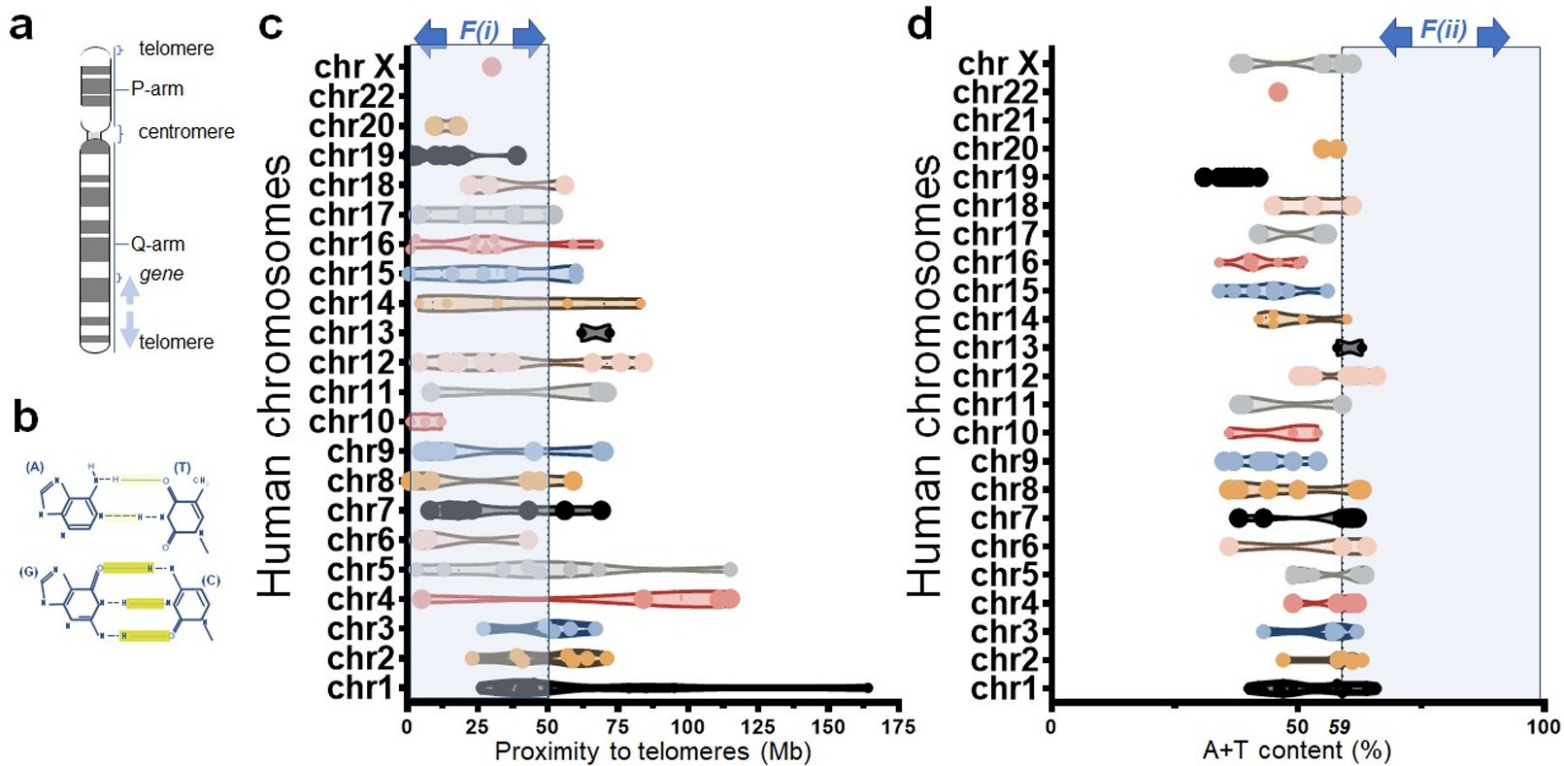
Genes encoding 129 druggable kinases with unknown disease associations. (a) a schematic diagram illustrating the relative positions of telomeres, centromere, each arm, and a gene in a human chromosome (b) illustration displaying two of four chemical bases comprising DNA, adenine and thymine whose combined (A+T) content is associated with high mutation rate in human chromosomes (c) Box and violin plots displaying the distribution of 129 kinases proposed as druggable proteins by the National Institute of Health (PA-19-034) with respect to proximity to telomeres over chromosome (chr) 1 to chr X. The candidate kinases are widely distributed over almost all chromosomes. F(i) indicates the proximity to the telomere. (d) Box and violin plots summarizing the full distribution of kinases over human chr 1 to chr X with respect to A+T content. F(ii) indicates high A+T content at >59%. Shaded rectangles below arrows indicate subsets of genes encoding kinases satisfying F(i) and F(ii) in a and b, respectively.

In assessing the list of the proposed genes encoding 129 kinases, we first surveyed F(i), the proximity of a gene to its telomere (**Fig 1A and 1B**). As we identified the transcripts of genes using the accession number assigned to each, we discovered the candidate genes encoding the 129 kinases of interest were widely distributed across almost all human chromosomes (**Fig 1C**). To assess the nucleotide compositions (F(ii)), we obtained the A+T content of each of the genes encoding these druggable kinases. These 129 kinase-encoding genes rarely expressed high A+T content at >59% (**Fig 1D**).

**Fig 2:**
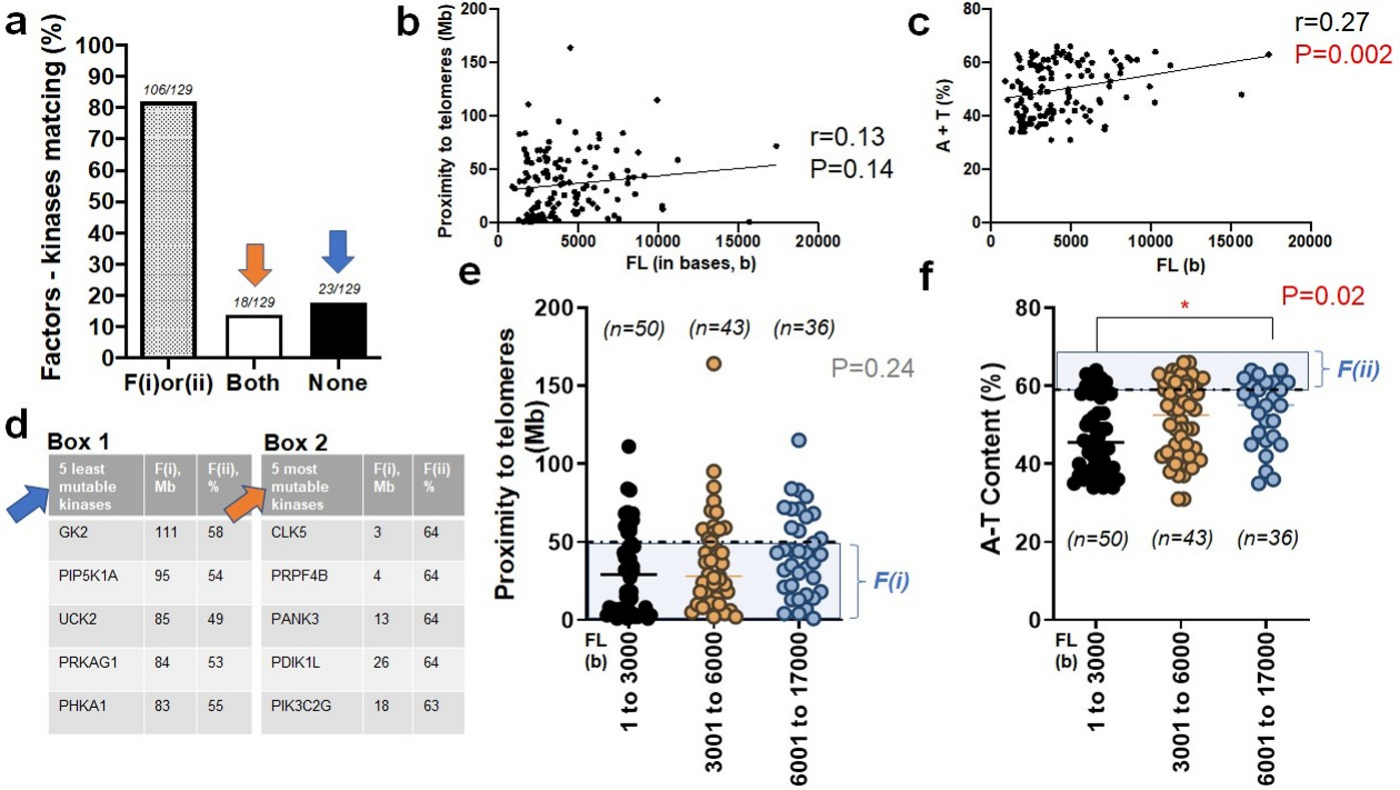
Prioritization of druggable kinases per the relative mutability. (a) Bar graph demonstrating the matching rate of genes encoding 129 kinases with two factors of telomere proximity, F(i), and nucleotide composition, F(ii). Genes sorted into three groups: those satisfying one of the two factors (F(i)or(ii)), those meeting F(i) and F(ii) (Both), and those satisfying neither F(i) nor F(ii) (None, blue arrow) (b) Scatter plot exhibiting the Pearson correlation between the full length of genes encoding 129 kinases and their proximity to telomeres. Pearson correlation coefficient at r = 0.13 with the level of statistical significance at P = 0.14. (c) Scatter plot showing the Pearson correlation between the full length of genes encoding 129 kinases and A+T content. Pearson correlation coefficient at r = 0.27 with the level of statistical significance at P = 0.002. (d) Five candidate genes (GK2, PIP5K1A, UCK2, PRKAG1, PHKA1) sorted per the relatively lower mutability (the ‘None’ group; Box 1); five alternative candidates (CKL5, PRPF4B, PANK3, PDIK1L, PIK3C2G) sorted per the relatively higher mutability (the ‘Both’ group; Box 2). (e) Scatter plot showing proximity to telomeres of genes encoding 129 kinases with respect to three sub-groups of the gene full-length size (unit: base or b). A horizontal dotted line indicates 50 Mb. No differences among three groups found at P = 0.24 after one-way ANOVA (f) Scatter plot summarizing A+T content of genes encoding 129 kinases with respect to the gene size in base or b. A horizontal dotted line indicates 59%. A significant difference between the shortest (1 to 3000 b) and longest size group (6001 to 17000 b) found at P = 0.02. F(i) and F(ii) indicate the first and second factor, respectively (e and f). *, P<0.05.

As we identified the matching rate between the two factors and the druggable kinases, 82% of genes satisfied either the proximity to telomeres or high A+T content (n = 106/129). More than 18% of genes (23 of 129) encoding druggable kinases met neither F(i) nor F(ii), while less than 15% of genes (18 of 129) met both F(i) and F(ii). Unlike the previous reports [23, 28], these two groups of “Both (meeting both factors)” and “None (meeting neither factor)” were particularly noteworthy as they implied relatively more and/or less mutable targets, in terms of the commercialization potential of drugs targeting these protein kinases (**Fig 2A**).

In examining the correlation between the molecular size and each factor, data entries suggested that the full-length size of a gene, if larger than 6,000 bases, was significantly correlated with the gene having high A+T content at >59%. Consistent with the prior report [23], a significant correlation was detected between the full-length size and A+T content (r = 0.27; P = 0.002) (**Fig 2B and 2C**). However, the Pearson coefficient (r = 0.13) indicated that there was no significant correlation (P = 0.14) between the full-length sizes of the genes and proximity to telomeres in druggable kinases.

Next, we examined the specific genes matching with ‘both’ or ‘none’ of the two factors and prioritized the top five kinases from each category. This allows us to filter out five candidate genes which showed relatively lower mutability (glycerol kinase 2 (GK2), phosphatidylinositol-4-phosphate 5-kinase type 1 alpha (PIP5K1A), uridine-cytidine kinase 2 (UCK2), protein kinase AMP-activated non-catalytic subunit gamma 1 (PRKAG1), and phosphorylase kinase regulatory subunit alpha 1 (PHKA1))—this corresponds to the ‘None’ group (blue arrow). Alternatively, five other candidate genes (CKL5, PRPF4B, PANK3, PDIK1L, and PIK3C2G) were sorted as the protein kinase genes with relatively higher mutability, corresponding to the ‘Both’ group (orange arrow; **Fig 2D**).

We next organized F(i) and F(ii) with respect to the nucleotide length. Following the previous analysis [23, 28], we grouped all genes into three categories: 1–3,000 bases (n = 50), 3,001–6,000 bases (n = 43), and 6,001–17,000 bases (n = 36), respectively. Statistical analysis suggested that there was no significant difference in proximity to telomeres with respect to the gene full-length size (P = 0.24). However, pair-wise comparisons using post-hoc test by Tukey’s after one-way ANOVA suggested that there was a significant difference (P = 0.02) in A+T content with respect to the full-length size (bases) between the shortest and longest subgroups (**Fig 2E and 2F**).

Our finding that there was a subset of kinase groups with a relatively low predicted mutability rate suggests that the best-selling kinase drugs on the market can fit into any of three categories meeting 1) one of the two factors or 2) both or 3) none.

### 73 cytokine genes found in the MIS-C met two factors at 85%

**Fig 3:**
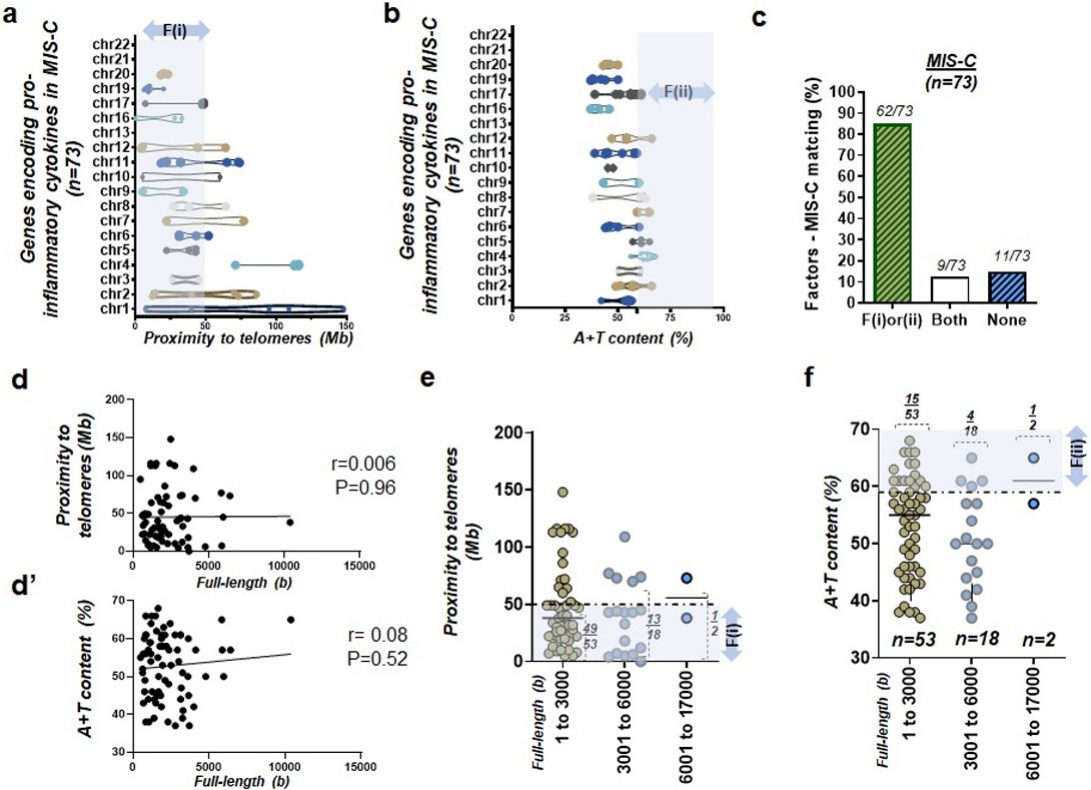
Genes (n = 73) encoding pro-inflammatory cytokines in MIS-C. (a) Box and violin plots displaying the distribution of genes encoding pro-inflammatory cytokines in MIS-C with respect to proximity to telomeres over chr 1 to 22. F(i) indicates distance to the telomere (b) Box and violin plots summarizing the full distribution of genes encoding pro-inflammatory cytokines with respect to A+T content over chr 1 to 22. F(ii) indicates A+T content if higher than 59%. (c) Bar graph demonstrating factors-disease (MIS-C) matching rate. Total number of genes, n = 73, with 85% match (62 of 73) of factor (i) or (ii) with MIS-C. (d) Scattered plot exhibiting the Pearson correlation between the full length (base or b) of genes encoding pro-inflammatory cytokines and their proximity to telomeres. Poor correlation (r = 0.006) with the level of statistical significance at P = 0.96. (D’) Scattered plot showing the Pearson correlation between the full length (base or b) of genes encoding pro-inflammatory cytokines and A+T content. Poor correlation (r = 0.08) with the level of statistical significance at P = 0.52. (e) Scatter plot showing proximity to telomeres of 73 genes encoding pro-inflammatory cytokines in MIS-C with respect to three sub-groups of the gene size by full-length in base or b. A horizontal dotted line indicates 50 Mb. The level of statistical difference after one-way ANOVA among three groups of 1 to 3000, 3001 to 6000, and 6001 to 17000 at P>0.05. (f) Scatter plot summarizing A+T content of genes encoding pro-inflammatory cytokines in MIS-C with respect to the gene size in base or b. A horizontal dotted line indicates 59%. The statistical difference after one-way ANOVA among three groups of 1 to 3000, 3001 to 6000, and 6001 to 17000 at P>0.05. F(i) and F(ii) indicate the first and second factor, respectively (a, b, e, and f).

As we investigated the list of the proposed genes encoding small-molecule ligands including binding sites for receptor tyrosine kinases, we examined the proximity to a telomere, F(i), in soluble protein ligands such as cytokines [33]. We identified the transcripts of genes using the assigned accession number and the genome data viewer, and found the candidate genes encoding 73 pro-inflammatory cytokines reported recently [9], similar to the kinase group, are widely distributed across almost all human chromosomes (**Fig 3A**).

To continue the assessment of the nucleotide compositions, we obtained the A+T content of genes encoding pro-inflammatory cytokines of the MIS-C. Consistent with the previous case of 129 protein kinases in the prior section, the genes encoding 73 pro-inflammatory cytokines which are highly responsive to the MIS-C rarely met high A+T content at >59% (**Fig 3B**). As we identified the matching rate between two factors and the pro-inflammatory cytokines differentially expressed in response to the MIS-C, 85% of 73 genes encoding pro-inflammatory cytokines satisfied either the proximity to telomeres or high A+T content (n = 62/73). Of note, approximately 12% of genes (9 of 73) encoding pro-inflammatory cytokines of the MIS-C met both F(i) and F(ii), whereas roughly 15% of genes (11 of 73) met neither F(i) nor F(ii) (**Fig 3C**).

In examining the correlation between the molecular size and each factor, the Pearson coefficient (r = 0.006) indicated that there was no significant correlation (P = 0.96) between the full-length sizes of the genes and proximity to telomeres in pro-inflammatory cytokines of the MIS-C. Unlike the protein kinases examined in the previous section, there was also no significant difference between the full-length size of the genes encoding pro-inflammatory cytokines and their A+T content (r = 0.08; P = 0.52) (**Fig 3D and 3D’**).

We then organized F(i) and F(ii) with respect to the nucleotide length. Following previous analyses [23, 28], we separated all genes into three categories: 1–3,000 bases (n = 53), 3,001–6,000 bases (n = 18), and 6,001–17,000 bases (n = 2). Statistical analysis suggested that there was no significant difference in proximity to telomeres with respect to the gene full-length size. Pair-wise comparisons using post-hoc test by Tukey’s after one-way ANOVA suggested that there was no significant difference in A+T content with respect to the full-length size (bases) (**Fig 3E and 3F**).

### 20 mutations due to ionizing radiation met two factors at only 50%

**Fig 4:**
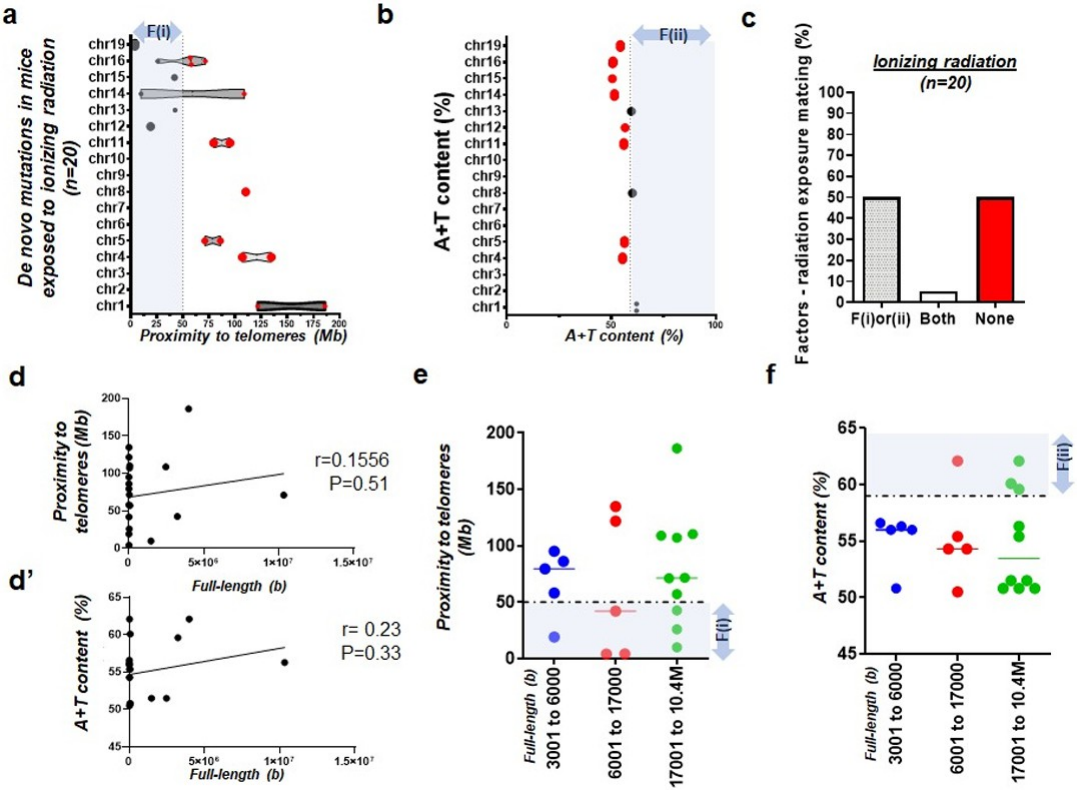
De novo mutations (n = 20) previously reported in mice after ionizing radiation. (a) Box and violin plots displaying the distribution of genetic loci with copy number variation (CNV) or de novo mutations in mice exposed to ionizing radiation with respect to proximity to telomeres over chr 1 to 19. F(i) indicates distance to the telomere (b) Box and violin plots summarizing the full distribution of genetic loci with de novo mutations in mice after ionizing radiation with respect to A+T content over chr 1 to 19. F(ii) indicates A+T content if higher than 59%. (c) Bar graph demonstrating factors-radiation matching rate. Total number of genes, n = 20, with 50% match (10 of 20) of factor (i) or (ii) with ionizing radiation. (d) Scattered plot exhibiting the Pearson correlation between the full length (base or b) of genetic loci of mice showing de novo mutations after ionizing radiation and their proximity to telomeres. Poor correlation (r = 0.1556) with the level of statistical significance at P = 0.51. (D’) Scattered plot showing the Pearson correlation between the full length (base or b) of genetic loci in mice and A+T content. Poor correlation (r = 0.23) with the level of statistical significance at P = 0.33. (e) Scatter plot showing proximity to telomeres of 20 genetic loci of mice showing de novo mutation after radiation with respect to three sub-groups of the gene size by full-length in base or b. A horizontal dotted line indicates 50 Mb. The level of statistical difference after one-way ANOVA among three groups of 3001 to 6000, and 6001 to 17000, and 17000 to 10.4 Mb at P>0.05. (f) Scatter plot summarizing A+T content of genetic loci of mice with de novo mutations with respect to the gene size in base or b. A horizontal dotted line indicates 59%. The statistical difference after one-way ANOVA among three groups of 3001 to 6000, and 6001 to 17000, and 17000 to 10.4 Mb at P>0.05. F(i) and F(ii) indicate the first and second factor, respectively (a, b, e, and f).

To compare the data above with the genomic characteristics of *de novo* mutations arising from environmental insults, we sought recent studies where the two factors, F(i) and F(ii), can be applied. We found that there were 20 loci of copy number variation (CNV) mutations reported in a wide range of mouse chromosomes [29]. Even though the CNVs reported in mice were widely distributed across almost all chromosomes, unlike the prior two cases of genes associated with kinases and cytokines, these *de novo* mutations failed to give rise to a high match between the two factors and the genetic loci when exposed to ionizing radiation (**Fig 4A and 4B**). Specifically, the matching and mismatch rates were identical at 50% (**Fig 4C**). Furthermore, there were no significant differences between the full-length size of the pertinent genetic loci and either of the two factors (**Fig 4D-4F**). These poor matching rates are consistent with findings in polygenic diseases caused by both genetic and environmental factors, in which the factor-disease matching rates were also equivalent at ~50%, as reported previously [23, 28].

### Approved drug target kinases/cytokines showed consistent features

To gain better understanding of genomic characteristics associated with mutations in novel druggable genomes with known commercialized drugs, we surveyed the mechanisms of action of top selling drugs whose target proteins are protein kinases or cytokines. First, a survey of FDA-approved commercialized drugs that target protein kinases identified three drugs, which inhibit EGFR, fibroblast growth factor receptor (FGFR), and platelet-derived growth factor receptor (PDGFR), as displayed in **Table 1**. For osimertnib, EGFR demonstrated a ‘marginal’ proximity to a telomere, consistent with transmembrane protein 67 (TMEM67), whose proximity to its telomere was slightly higher than ≅ 50Mb, as reported previously [23, 28]. For erdafitinib, FGFR satisfied the proximity to its telomere at 38 Mb. For imatinib, PDGFR similarly satisfied proximity to its telomere at 31 Mb. Moreover, these three drugs showed that their target proteins have A+T content that are less than the human genome-wide average of 59% (**Table 1**).

**Table 1:** Two factor characteristics of protein kinases targeted by the FDA-approved drugs.

Generic name	Gene	Chr*	Gene locus (Mb)	Telomere locus (Mb)	Gene to telomere (Mb)	A,T (%)	A+T (%)	Length (bp)	Market size	Indication	Ref
Osimertinib	EGFR	7	55	0	55	27,29	56	9905	$4.3 billion (2020)	non-small cell lung cancer	[34]
Erdafitinib	FGFR	8	38	0	38	22,23	45	5691	$1.15 million (2024)	Bladder cancer	[35]
Imatinib	PDGFR	5	150	181	31	21,22	43	5700	$2.5 billion (2015)	Philadelphia chromosome-positive chronic myeloid leukemia	[1]

* Chr, chromosome; Ref, reference

In a similar fashion, the measurements of genomic characteristics on cytokines targeted by the FDA-approved commercially active drugs demonstrated that five different genes encoding tissue necrosis factor (TNF), interleukin 17-A (IL-17A), interleukin 6 (IL-6), growth differentiation factor 8 (GDF8), and vascular endothelial growth factor (VEGF) satisfied either proximity to telomeres at <50 Mb or high A+T content at >59%, whereas guselkumab—which targets interleukin 23 (IL-23)—satisfied neither factor (**Table 2**).

**Table 2:** Two factor characteristics of cytokines targeted by the FDA-approved drugs.

Generic name	Gene	Chr*	Gene locus (Mb)	Telomere locus (Mb)	Gene to telomere (Mb)	A,T (%)	A+T (%)	Length (bp)	Market size	Indication	Ref
Golimumab	TNF	6	31	0	31	23,23	56	1678	$39 billion (2020)	active psoriatic arthritis	[33]
Secukinumab	IL-17A	6	52	0	52	30,30	60	1871	$3.9 billion (2020)	active psoriatic arthritis.	[33, 36]
Siltuximab	IL-6	7	22	0	22	30,29	59	1127	Some portion of $20.7 billion (2018)	multicentric Castleman’s disease	[33]
Guselkumab	IL-23	12	56	132	76	25,25	50	1037	Some portion of $143 million (2021)	moderate-to-severe plaque psoriasis	[33]
Luspatercept	GDF8	2	190	241	51	34,33	67	2819	$115 million (2020)	anemia	[33]
Bevacizumab	VEGF	6	43	0	43	26,26	52	3609	$30 million (2021)	non-small cell lung cancer /Eye disease	[33]

* Chr, chromosome; Ref, reference

## Discussion

Causative mutations that result in human diseases are commonly regarded to be inherited from one’s parents through the germline and are detected in somatic cells, except for the majority of cancer mutations, which ensue somatically. Mounting evidence suggests that somatic mutations are present not only in cancer, but also both adult cardiovascular diseases [37, 38], and newborn neurological disorders [39]. The mutations that are detected in the sperm or egg of a parent but are not present in their blood are considered *de novo* mutations. Thus, *de novo* mutations are found in affected offspring but not present in the parents, and are often associated with neuropsychiatric and pediatric disorders [39]. Although the two factors that we applied to genes encoding protein kinases and pro-inflammatory cytokines do not specify how these two factors alone can selectively affect germline, somatic, and/or *de novo* mutations, our analysis on the approved EGFR inhibitor drugs suggests that the threshold for F(i) or proximity to telomeres should be corrected from 50 to 77 Mb [23, 28]—at least for somatic mutations in cancer—to embrace marginal proximity conditions to meet the condition of sufficiently proximal distance to telomeres.

Our evaluations of cytokines suggest that genes actively play a role in host defenses and are therefore more highly responsive to external stimuli such as viral infections, meaning that they are also more likely to harbor genomic characteristics with high mutability. Similarly, the genomic analyses on receptor tyrosine kinases suggest that genes which are highly activated in response to binding with ligands, like EGFR signaling in cancer, have evolved in a way to be mutable—as measured by proximity to telomeres and A+T content.

Three genomic factors in addition to others [27] were reported to be associated with high mutation rates, including recombination rate, proximity to telomeres, and high A+T content [24]. Among these factors, we have applied two of the factors to druggable kinases to demonstrate the mutability of these proteins. Our data reveal that 129 of the understudied kinases proposed as druggable proteins by the IDG are highly susceptible to germline or somatic mutations. The results suggest that 82% of these druggable kinases are prone to a high mutation rate, due primarily to their proximity to telomeres and/or high A+T content, and that 12 of 129 kinases (18%) meet neither F(i) nor F(ii). As such, this ~18% of protein kinases, if used as drug targets, would be expected to be less likely to mutate.

Our systematic analysis of the 129 druggable protein kinases (NIH PA-19-034) resulted in a profile consistent with the previous reports on genetic loci associated with human genetic and age-related diseases [23, 28], demonstrating that the candidate genes are distributed throughout all of the human chromosomes. Furthermore, the idea that a poor match rate indicated that the disease was polygenic, while a higher match rate implied the disease was monogenic, supports our observation that germline or somatic mutations caused by genetic factors alone or by the mixed effects of both genetic and environmental factors can be predicted by the matching rate analysis using these two factors. On the other hand, *de novo* mutations arising from environmental factors such as ionizing radiation cannot be explained completely, as indicated by a low match at ~50%, suggesting additional factors^17,18^ should be taken into consideration.

The factor-kinase matching rate (**Fig 1A and 1B**), the distributions of each factor over the molecular sizes of the target nucleotide (**Fig 2B and 2C**), and the statistical comparison of the factor-molecular size (**Fig 2E and 2F**) suggest that the 129 protein kinases studied have longer molecular sizes than cytokines, since roughly 20 times more target molecules (n = 36 at 6,001–17,000 bases group; **Fig 2F**) have full-length sizes between 6,001–17,000 bases than that of the cytokines group (n = 2 at 6,001–17,000 bases group; **Fig 3F**). Consistent with the prior reports on genes associated with high mutation rates in human diseases [23, 28], there was a statistical significance between the molecular size of the genes encoding protein kinases and A-T content (**Fig 2C and 2F**), while no significant difference was detected in the genes encoding small-molecules or cytokines (**Fig 3C and 3F**). Such a difference in size between the protein kinases (relatively larger) and cytokines (relatively smaller) echoes the previous finding that the relationship between the full-length size of the genes under analysis and the A+T content is positively correlated and that the longer size groups—the 3,001 to 6,000 bases group and the 6,001 to 17,000 bases group compared to 1 to 3,000 bases group—is the key to determining the statistical significance [23, 28].

Advances in molecular and cellular biology, genomics, and pharmacology in the past few decades have also shifted the paradigm for therapeutics from relatively large molecules to small ones, resulting in several other therapeutic modalities—including full-length monoclonal antibodies (mAbs). Owing to this antibody-based therapy, small-molecules such as soluble factors or inflammatory cytokines have become an increasingly significant class of drug targets. Cytokines and/or growth factors are smaller molecules than kinases and constitute 50% of the 22 ligands targeted by FDA-approved drugs and 40% of the 77 novel ligands for which agents are under development [33]. More than 80 mAbs have been approved since the pioneering mAb, muromonab-CD3, was developed in 1986, and three of the best-selling drugs in 2018 and 2019 were ligand-targeting mAbs [33]. Soluble ligands, which are small in molecular sizes compared to receptor kinases, do not appear to have an issue with mutations as druggable targets except at the ligand binding site [8, 33]. However, their binding partners (ligand receptors) and downstream signaling molecules are mutable in both cancer and non-cancerous conditions [1].

Soluble ligands potentiate mutations in their ligand binding sites [8] and affect mutations of downstream molecules involved in the MAP kinase cascade [1]. Cytokines mediate inflammation and actively respond to viral mutations as shown in recent cases of COVID-19 [9]. The B.1.617.2 (Delta) variant of COVID-19 has caused a serious problem in re-escalating the infection rate globally due primarily to the high ‘transmission rate’ even post vaccine administration [10]. When multisystem inflammatory syndrome in children (MIS-C) [9] first became an issue, the COVID-19 vaccines had not yet been released and adults comprised the majority of confirmed cases [11]. It was thought that the reason children have fared well against COVID-19 could lie in the innate immune response–the body’s crude but swift reaction to pathogens. For now, there is no clear evidence that children are more vulnerable to or more affected by Delta in comparison with earlier variants. Like all viruses, SARS-Cov-2 is constantly mutating and becoming better at evading host defenses, and that makes understanding the additional protective benefits seen in children important to examine [11].

The limitation of applying two factors to genes associated with rare circumstances can be found in genetic diseases that would not be discerned by the advanced sequencing techniques. One of the relevant examples is Friedreich’s ataxia (FA) [40], an inherited neurodegenerative disorder that affects the nervous system with debilitating symptoms affecting movements and reflexes resulting from impaired mitochondrial function. The cause of FA is mostly due to abnormal repetitions of the triplet repeat of the nucleotide sequence GAA in the frataxin (FXN) gene encoding the mitochondrial protein frataxin. In this case, measuring WT/unaffected sequences of the FXN gene fails to meet the two factors, although the mutant FXN gene satisfies the second factor, F(ii). This trinucleotide repeat causes gene silencing, in which the FXN gene is not transcribed normally [41]. As a result, a reduced level of frataxin protein is made in patients with FA. In this case, a sustained expression of the WT FXN through gene therapy might augment restoration of neurodegenerative symptoms back to normal. Since there is a set of previous reports [42–61] defining a role for phosphatases such as Protein phosphatase 2A (PP2A) as a negative regulator of protein kinase function and activation, future studies should warrant the potential consequences of PP2A [50] or similar phosphatase function in the absence or presence of mutations in the 129 genes encoding protein kinases investigated in this study.

## Conclusion

Among the 129 druggable kinases, 106 human genes encoding protein kinases satisfied either proximity to telomeres at <50 Mb or high A+T content at >59%, suggesting 82% of these genes are mutable. Of 73 genes encoding pro-inflammatory cytokines of the MIS-C, 62 human genes encoding these cytokines met either F(i) or F(ii), suggesting that 85% of these genes have high mutability. Mice exposed to space-like ionizing radiation give rise to offspring with 20 de novo mutations, resulting in only 10 of these 20 murine genetic loci met (i) or (ii), leading to only a 50% match. When compared with the mechanisms of top-selling FDA approved drugs, this data suggests that matching rate analysis on druggable targets is feasible to systematically prioritize the relative mutability—and therefore therapeutic potential—of the novel candidates.

## Supporting information

S1 Table Two factor characteristics of 129 druggable kinases. * chr, chromosome; ** A, adenine; T, thymine; *** FL, full-length; bp, base pair.
(DOCX)

S2 Table Two factor characteristics of 73 pro-inflammatory cytokines of the MIS-C*. * Multisystem inflammatory syndrome in children, MIS-C.
(DOCX)

S3 Table Two factor characteristics of druggable kinases and select approved drugs. * Not significant, NS; ** significant, Sig.
(DOCX)

S4 Table Two factor characteristics of genetic loci in mice exposed to ionizing radiation. * Copy number variation or de novo mutations found in mice after ionizing radiation2, CNV; ** A+T content of the entire chromosome calculated.
(DOCX)
