# Supplementary material for: Mutability of druggable kinases and pro-inflammatory cytokines by their proximity to telomeres and A+T content
Source: PLoS One. 2023 Apr 27;18(4):e0283470. doi: 10.1371/journal.pone.0283470 (PMC10138820; doi:10.1371/journal.pone.0283470)
Supplement: S1 Table — * chr, chromosome; ** A, adenine; T, thymine; *** FL, full-length; bp, base pair. (DOCX) [file pone.0283470.s001.docx]

Supporting Information

Mutability of druggable kinases and pro-inflammatory cytokines by their proximity to telomeres and A+T content

Ian McKnight^1^, Regan Raines^1^, Hunter White^1^,

Nasim Nosoudi^1^, Chan Lee^2^, Peter H.U. Lee^3,4^, Joon W. Shim^1^,*

Correspondence to: [shim@marshall.edu](mailto:shim@marshall.edu)

**This file includes:**

S1 Table

**S1 Table. Two factor characteristics of 129 druggable kinases.**

| Kinases | gene ID | chr* | gene locus | telomere locus | gene to telomere | A, T ** (%) | A + T (%) | FL*** (bp) |
| --- | --- | --- | --- | --- | --- | --- | --- | --- |
| 1 | PRKAG1 | 12 | 49 Mb | 133 Mb | 133-49 = 84 | 25,28 | 53% | 1657 |
| 2 | PRKAB1 | 12 | 119 Mb | 133 Mb | 133-119 = 14 | 23,27 | 50% | 2219 |
| 3 | ADCK5 | 8 | 144 Mb | 145 Mb | 145 – 144 = 1 | 17,19 | 36% | 1960 |
| 4 | ALPK3 | 15 | 84 Mb | 100 Mb | 100 – 84 = 16 | 22,23 | 45% | 10238 |
| 5 | ADCK4 | 19 | 40 Mb | 58 Mb | 58 - 40 = 18 | 18, 20 | 38 | 2443 |
| 6 | ALPK2 | 18 | 58 Mb | 80 Mb | 80 - 58 = 22 | 30, 23 | 53 | 7437 |
| 7 | BCKDK | 16 | 31 Mb | 0 | 31 | 19, 21 | 40 | 2036 |
| 8 | CAMKV | 3 | 49 Mb | 0 | 49 | 21,22 | 43 | 2998 |
| 9 | CDKL3 | 5 | 134 Mb | 181 Mb | 181-134=47 | 34,28 | 62 | 1717 |
| 10 | CDKL4 | 2 | 39 Mb | 0 | 39 | 33,30 | 63 | 1623 |
| 11 | CDKL1 | 14 | 50 Mb | 107 Mb | 107 - 50=57 | 29,31 | 60 | 5402 |
| 12 | CDKL2 | 4 | 75 Mb | 190 Mb | 190-75 =115 | 30,32 | 62 | 4704 |
| 13 | CDK15 | 2 | 201 Mb | 242 Mb | 242-201=41 | 29,29 | 58 | 3587 |
| 14 | CDK17 | 12 | 96 MB | 133 Mb | 133-96=37 | 28,32 | 60 | 4036 |
| 15 | CDK14 | 7 | 90 Mb | 159 Mb | 159-90=69 | 29,32 | 61 | 4970 |
| 16 | CDK18 | 1 | 205 Mb | 248 Mb | 248-205=43 | 20,20 | 40 | 3068 |
| 17 | CLK4 | 5 | 178 Mb | 181 Mb | 181-178=3 | 32,32 | 64 | 2504 |
| 18 | CDK10 | 16 | 89 Mb | 90 Mb | 90-89=1 | 19,22 | 41 | 1595 |
| 19 | CLK3 | 15 | 74 Mb | 101 Mb | 101-74=27 | 24,21 | 45 | 1846 |
| 20 | CSNK2A2 | 16 | 58 Mb | 90 Mb | 90-58=32 | 24,22 | 46 | 1887 |
| 21 | DGKH | 13 | 42 Mb | 114 Mb | 114-42=72 | 30,33 | 63 | 17366 |
| 22 | DYRK3 | 1 | 206 Mb | 248 Mb | 248-206=42 | 26,32 | 58 | 8128 |
| 23 | DYRK1B | 19 | 39 Mb | 58 Mb | 58-39=19 | 17,20 | 37 | 2496 |
| 24 | DYRK4 | 12 | 4 Mb | 0 Mb | 4-0=4 | 28,24 | 52 | 1845 |
| 25 | EEF2K | 16 | 22 Mb | 90 Mb | 90-22=68 | 23,28 | 51 | 7398 |
| 26 | DYRK2 | 12 | 67 Mb | 133 Mb | 133-67=66 | 28,33 | 61 | 8739 |
| 27 | ERN2 | 16 | 23 Mb | 0 Mb | 23-0=23 | 20,20 | 40 | 3337 |
| 28 | FASTK | 7 | 151 Mb | 159 Mb | 159-151=8 | 16,22 | 38 | 1794 |
| 29 | GK2 | 4 | 79 MB | 190 Mb | 190-79=111 | 29,29 | 58 | 1868 |
| 30 | HIPK4 | 19 | 40 MB | 58 MB | 58-40=18 | 19,20 | 39 | 2446 |
| 31 | ITPKA | 15 | 41 Mb | 101 Mb | 101-41=60 | 17,17 | 34 | 1825 |
| 32 | CSNK1G3 | 5 | 123 Mb | 181 Mb | 181-123=58 | 30,33 | 63 | 4360 |
| 33 | CSNK1G2 | 19 | 1.9 MB | 0 Mb | 1.9-0=1.9 | 19,17 | 36 | 2895 |
| 34 | PRKACG | 9 | 69 Mb | 138 MB | 138-69=69 | 22,22 | 44 | 1610 |
| 35 | CSNK1G1 | 15 | 64 Mb | 101 M b | 101-64=37 | 27,29 | 56 | 8085 |
| 36 | ITPK1 | 14 | 93 MB | 107 Mb | 107-93=14 | 19,23 | 42 | 6188 |
| 37 | PSKH2 | 8 | 86 Mb | 145 M b | 145-86=59 | 31,31 | 62 | 2557 |
| 38 | CAMK1D | 10 | 12 Mb | 0 MB | 12-0=12 | 26,23 | 49 | 2085 |
| 39 | TK2 | 16 | 66 MB | 90 MB | 90-66=24 | 22,28 | 50 | 4824 |
| 40 | PRKACB | 1 | 84 MB | 248 MB | 248-84=164 | 29,35 | 64 | 4496 |
| 41 | PNCK | X | 153 Mb | 156 Mb | 156-153=3 | 19,20 | 39 | 1888 |
| 42 | CAMK1G | 1 | 209 Mb | 249 Mb | 249-209=40 | 23,24 | 47 | 2456 |
| 43 | CAMKK1 | 17 | 3.8 Mb | 0 Mb | 3.8-0=3.8 | 21,21 | 42 | 3572 |
| 44 | PSKH1 | 16 | 67 Mb | 90 Mb | 90-67=28 | 18,23 | 41 | 3497 |
| 45 | PHKA1 | X | 72 Mb | 155 Mb | 155-72=83 | 26,29 | 55 | 6286 |

***** chr, chromosome; ** A, adenine; T, thymine; *** FL, full-length; bp, base pair

**S1 Table (continued)**

| Kinases | gene ID | chr | gene locus | telomere locus | gene to telomere | A, T (%) | A + T (%) | FL (bp) |
| --- | --- | --- | --- | --- | --- | --- | --- | --- |
| 46 | RPS6KC1 | 1 | 213 Mb | 248 Mb | 248-213=35 | 29,30 | 59 | 5505 |
| 47 | PRKCQ | 10 | 6.3 MB | 0 Mb | 6.3-0=6.3 | 28,26 | 54 | 3255 |
| 48 | LMTK3 | 19 | 48 Mb | 58 Mb | 58-48=10 | 16,15 | 31 | 4945 |
| 49 | MARK4 | 19 | 45 Mb | 58 Mb | 58-45=13 | 21,21 | 42 | 5231 |
| 50 | MAST3 | 19 | 18 Mb | 0 Mb | 18-0=18 | 19,21 | 40 | 5906 |
| 51 | LRRK1 | 15 | 101 Mb | 101.5 Mb | 0.5 | 23,25 | 48 | 15674 |
| 52 | MAP3K14 | 17 | 45 Mb | 83 Mb | 83-45=38 | 22,20 | 42 | 4442 |
| 53 | LTK | 15 | 41 Mb | 101 Mb | 101-41=60 | 17,20 | 37 | 3072 |
| 54 | MAP3K10 | 19 | 40 Mb | 58 Mb | 58-40=18 | 16,15 | 31 | 3754 |
| 55 | MAST4 | 5 | 66 Mb | 181 Mb | 181-66=115 | 26,25 | 51 | 9918 |
| 56 | MAST2 | 1 | 45 Mb | 0 Mb | 45-0=45 | 22,21 | 43 | 5737 |
| 57 | MAPK15 | 8 | 143 MB | 145 Mb | 145-143=2 | 17,19 | 36 | 1871 |
| 58 | MAPK4 | 18 | 50 Mb | 79 Mb | 79-50=29 | 23,22 | 45 | 4763 |
| 59 | MKNK2 | 19 | 2 Mb | 0 Mb | 2-0=2 | 20,20 | 40 | 1758 |
| 60 | CDC42BPG | 11 | 64 Mb | 135 Mb | 135-64=71 | 20,18 | 38 | 6161 |
| 61 | CDC42BPB | 14 | 103 Mb | 107 Mb | 107-103=4 | 24,21 | 45 | 6844 |
| 62 | NEK11 | 3 | 131 Mb | 198 Mb | 198-131=67 | 31,26 | 57 | 2926 |
| 63 | NEK5 | 13 | 52 Mb | 114 Mb | 114-52=62 | 31,27 | 58 | 2913 |
| 64 | NEK10 | 3 | 27 Mb | 0 Mb | 27-0=27 | 31,31 | 62 | 8543 |
| 65 | NIM1K | 5 | 43 MB | 0 Mb | 43-0=43 | 27,22 | 49 | 2313 |
| 66 | NEK4 | 3 | 52 Mb | 0 Mb | 52-0=52 | 27,30 | 57 | 6053 |
| 67 | NEK6 | 9 | 124 Mb | 138 Mb | 138-124=14 | 24,25 | 49 | 3468 |
| 68 | NRK | X | 105 MB | 154 Mb | 154-105=49 | 31,28 | 59 | 8066 |
| 69 | NEK7 | 1 | 198 Mb | 248 Mb | 248-198=50 | 30,36 | 66 | 4114 |
| 70 | SCYL1 | 11 | 65 MB | 133 MB | 133-65=68 | 21,18 | 39 | 2636 |
| 71 | NRBP2 | 8 | 143 Mb | 145 Mb | 145-143=2 | 22,22 | 44 | 3724 |
| 72 | PIK3C2G | 12 | 18 Mb | 0 Mb | 18-0=18 | 32,31 | 63 | 4864 |
| 73 | PAK7 | 20 | 9.5 Mb | 0 Mb | 9.5-0=9.5 | 29,26 | 55 | 4834 |
| 74 | PANK3 | 5 | 168 MB | 181 Mb | 181-168=13 | 30,34 | 64 | 10274 |
| 75 | PIK3C2B | 1 | 204 Mb | 247 Mb | 247-204=43 | 21,26 | 47 | 8457 |
| 76 | PAK3 | X | 111 Mb | 155 MB | 155-111=44 | 30,31 | 61 | 9126 |
| 77 | PAK6 | 15 | 40 Mb | 99 Mb | 99-40=59 | 20,21 | 41 | 3872 |
| 78 | SCYL3 | 1 | 169 Mb | 248 Mb | 248-169=79 | 30,30 | 60 | 6308 |
| 79 | PDIK1L | 1 | 26 Mb | 0 Mb | 26-0=26 | 31,33 | 64 | 4113 |
| 80 | PIP4K2C | 12 | 57 Mb | 133 Mb | 133-57=76 | 22,29 | 51 | 3176 |
| 81 | PIP5K1A | 1 | 151 Mb | 246 Mb | 246-151=95 | 24,30 | 54 | 3761 |
| 82 | PLK5 | 19 | 1.5 Mb | 0 Mb | 1.5 | 19,20 | 39 | 3760 |
| 83 | PHKG1 | 7 | 56 Mb | 0 Mb | 56 | 23,20 | 43 | 2074 |
| 84 | PI4KA | 22 | 20 Mb | 50 Mb | 30 | 22,24 | 46 | 6751 |
| 85 | PIP5K1B | 9 | 68 MB | 138 Mb | 138-68=70 | 27,27 | 54 | 3115 |
| 86 | PKMYT1 | 16 | 2.9 Mb | 0 Mb | 2.9 | 15,19 | 34 | 2061 |
| 87 | PKN3 | 9 | 128 Mb | 138 Mb | 10 | 18,19 | 37 | 3393 |
| 88 | PRPF4B | 6 | 4 Mb | 0 Mb | 4 | 32,32 | 64 | 7517 |
| 89 | TP53RK | 20 | 46 Mb | 64 Mb | 18 | 25,33 | 58 | 3180 |
| 90 | PRKRA | 2 | 242 Mb | 178 Mb | 242-178=64 | 30,28 | 58 | 1770 |

**S1 Table (continued)**

| Kinases | gene ID | chr | gene locus | telomere locus | gene to telomere | A, T (%) | A + T (%) | FL (bp) |
| --- | --- | --- | --- | --- | --- | --- | --- | --- |
| 91 | PRKY | Y | 7.2 MB | 0 Mb | 7.2 | 27,28 | 55 | 7240 |
| 92 | PXK | 3 | 58 Mb | 0 MB | 58-0=58 | 29,29 | 58 | 3001 |
| 93 | RIOK1 | 6 | 7.3 Mb | 0 Mb | 7.3 | 31,28 | 59 | 2498 |
| 94 | RIOK3 | 18 | 23 Mb | 79 MB | 79-23=56 | 31,30 | 61 | 3574 |
| 95 | RPS6KL1 | 14 | 74 Mb | 106 Mb | 32 | 22,23 | 45 | 5322 |
| 96 | SBK2 | 19 | 55 MB | 58 Mb | 3 | 15,22 | 37 | 2251 |
| 97 | SCYL2 | 12 | 100 Mb | 133 Mb | 33 | 30,33 | 63 | 5977 |
| 98 | SBK3 | 19 | 55 Mb | 58 MB | 3 | 17,17 | 34 | 1270 |
| 99 | SGK494 | 17 | 28 MB | 80 MB | 52 | 25,31 | 56 | 3772 |
| 100 | POMK | 8 | 43 Mb | 0 Mb | 43 | 23,27 | 50 | 1868 |
| 101 | SGK223 | 8 | 8.3 Mb | 0 Mb | 8.3 | 20,18 | 38 | 4845 |
| 102 | SRPK3 | X | 153 Mb | 156 Mb | 3 | 20,18 | 38 | 1960 |
| 103 | STK32C | 10 | 132 Mb | 133 Mb | 1 | 18,18 | 36 | 2096 |
| 104 | STK33 | 11 | 8.3 Mb | 0 Mb | 8.3 | 31,28 | 59 | 2728 |
| 105 | STK32B | 4 | 5 MB | 0 Mb | 5 | 24,25 | 49 | 3535 |
| 106 | STK40 | 1 | 36 Mb | 0 Mb | 36 | 20,22 | 42 | 3882 |
| 107 | STK17A | 7 | 43 Mb | 0 Mb | 43 | 30,32 | 62 | 3918 |
| 108 | STK36 | 2 | 241 Mb | 218 Mb | 241-218=23 | 21,26 | 47 | 4873 |
| 109 | STK32A | 5 | 147 Mb | 181 Mb | 181-147=34 | 27,26 | 53 | 878 |
| 110 | STK38L | 12 | 27 Mb | 0 Mb | 27-0=27 | 32,34 | 66 | 4957 |
| 111 | STKLD1 | 9 | 133 Mb | 138 MB | 138-133=5 | 21,23 | 44 | 2826 |
| 112 | STK3 | 8 | 98 Mb | 145 Mb | 145-98=47 | 32,31 | 63 | 2833 |
| 113 | STK31 | 7 | 23 Mb | 0 Mb | 23 | 32,29 | 61 | 3274 |
| 114 | TESK2 | 1 | 45 Mb | 0 MB | 45 | 23,26 | 49 | 3071 |
| 115 | TBCK | 4 | 106 Mb | 190 Mb | 190-106=84 | 30,31 | 61 | 7772 |
| 116 | TLK1 | 2 | 171 MB | 242 MB | 242-171=71 | 30,31 | 61 | 5723 |
| 117 | TPK1 | 7 | 144 Mb | 159 MB | 159-144=15 | 29,31 | 60 | 2439 |
| 118 | TLK2 | 17 | 62 Mb | 83 MB | 83-62=21 | 28,27 | 55 | 5640 |
| 119 | TSSK6 | 19 | 19 MB | 58 MB | 58-19=39 | 17,18 | 35 | 1330 |
| 120 | TSSK1B | 5 | 113 MB | 181 Mb | 181-113=68 | 25,24 | 49 | 2437 |
| 121 | TTBK2 | 2 | 42 Mb | 101 Mb | 101-42=59 | 29,30 | 59 | 11208 |
| 122 | TSSK4 | 14 | 24 Mb | 107 Mb | 107-24=83 | 25,26 | 51 | 1317 |
| 123 | TTBK1 | 6 | 43 Mb | 0 Mb | 43 | 19,17 | 36 | 7130 |
| 124 | TSSK3 | 1 | 32 MB | 0 Mb | 32 | 25,21 | 46 | 1026 |
| 125 | UCK1 | 9 | 131 MB | 138 Mb | 138-131=7 | 21,21 | 42 | 2162 |
| 126 | UCK2 | 1 | 165 Mb | 248 Mb | 248-165=85 | 22,27 | 49 | 4801 |
| 127 | WNK2 | 9 | 93 MB | 138 Mb | 138-93=45 | 18,17 | 35 | 7080 |
| 128 | VRK2 | 2 | 57 MB | 0 Mb | 57-0=57 | 33,28 | 61 | 1773 |
| 129 | WEE2 | 7 | 141 Mb | 159 Mb | 159-141=18 | 29,30 | 59 | 3061 |
