# Supplementary material for: Mutability of druggable kinases and pro-inflammatory cytokines by their proximity to telomeres and A+T content
Source: PLoS One. 2023 Apr 27;18(4):e0283470. doi: 10.1371/journal.pone.0283470 (PMC10138820; doi:10.1371/journal.pone.0283470)
Supplement: S2 Table — * Multisystem inflammatory syndrome in children, MIS-C. (DOCX) [file pone.0283470.s002.docx]

Supporting Information

Mutability of druggable kinases and pro-inflammatory cytokines by their proximity to telomeres and A+T content

Ian McKnight^1^, Regan Raines^1^, Hunter White^1^,

Nasim Nosoudi^1^, Chan Lee^2^, Peter H.U. Lee^3,4^, Joon W. Shim^1^,*

Correspondence to: [shim@marshall.edu](mailto:shim@marshall.edu)

**This file includes:**

S2 Table

**S2 Table. Two factor characteristics of 73 pro-inflammatory cytokines of the MIS-C***

| Cytokines[1] | gene ID | chr | gene locus | telomere locus | gene to telomere | A, T (%) | A + T (%) | FL (bp) |
| --- | --- | --- | --- | --- | --- | --- | --- | --- |
| 1 | IL-18 | 11q | 112Mb | 134 Mb | 134-112=22 | 32, 27 | 59 | 1115 |
| 2 | IL-6 | 7p | 22Mb | 0 Mb | 22-0=22 | 30, 29 | 59 | 1127 |
| 3 | CCL-3 | 17q | 36Mb | 83 Mb | 83-36=47 | 21,28 | 49 | 780 |
| 4 | CCL-4 | 17q | 36Mb | 82 Mb | 82-36=47 | 23,31 | 56 | 660 |
| 5 | CDCP1 | 3p | 45Mb | 0 Mb | 45-0=45 | 24,26 | 50 | 5963 |
| 6 | IL-17A | 6p | 52Mb | 0 Mb | 52-0=52 | 30,30 | 60 | 1871 |
| 7 | CCL20 | 2q | 227 Mb | 241 Mb | 241-227=14 | 31,35 | 66 | 834 |
| 8 | CCL28 | 5p | 43 Mb | 0 | 43-0=43 | 28,33 | 61 | 3126 |
| 9 | HGF | 7q | 81 Mb | 158 Mb | 158-81=77 | 32,33 | 65 | 5834 |
| 10 | CXCL10 | 4q | 76 Mb | 189 Mb | 189-76=113 | 32,32 | 64 | 1175 |
| 11 | FGF-21 | 19q | 48 Mb | 58 Mb | 58-48-10 | 16,22 | 38 | 2292 |
| 12 | IFN-gamma | 12q | 68 Mb | 132 Mb | 132-68=64 | 33,33 | 66 | 1211 |
| 13 | CCL19 | 9p | 34 Mb | 0 | 34-0=34 | 22,21 | 43 | 683 |
| 14 | OPG | 8q | 118 Mb | 145 Mb | 145-118=27 | 32,29 | 61 | 2087 |
| 15 | LIF-R | 5p | 38 Mb | 0 | 38-0=38 | 30,35 | 65 | 10385 |
| 16 | CSF-1 | 1p | 109 Mb | 0 | 109-0=109 | 21,21 | 42 | 3994 |
| 17 | CCL23 | 17q | 36 Mb | 83 Mb | 83-36=47 | 25,26 | 51 | 628 |
| 18 | CX3CL1 | 16q | 57 Mb | 90 Mb | 90-57=33 | 19 22 | 41 | 3285 |
| 19 | PD-L1 | 9p | 5 Mb | 0 | 5-0=5 | 28 32 | 60 | 3634 |
| 20 | EN-RAGE | 1q | 153 Mb | 248 Mb | 248-153=95 | 28 27 | 55 | 485 |
| 21 | CXCL9 | 4q | 76 Mb | 189 | 189-76=113 | 30 31 | 61 | 2761 |
| 22 | IL-10 | 1q | 206 Mb | 246 Mb | 246-206=40 | 27 28 | 55 | 1630 |
| 23 | MCP-3 | 17q | 34 Mb | 83 | 83-34=49 | 30 31 | 61 | 810 |
| 24 | MCP-2 | 17q | 34 Mb | 83 | 83-34=49 | 29 32 | 61 | 862 |
| 25 | OSM | 22q | 30 Mb | 50 Mb | 50-20=30 | 20 22 | 42 | 1865 |
| 26 | TNFSF14 | 19p | 6 Mb | 0 | 6-0=6 | 27 23 | 50 | 4778 |
| 27 | TGF-alpha | 2p | 70 Mb | 0 | 70-0=70 | 26 31 | 57 | 4117 |
| 28 | IL8 | 4q | 73 Mb | 144 | 144-73=71 | 33 35 | 68 | 1642 |
| 29 | TGF b1 | 19q | 41 Mb | 51 Mb | 51-41=10 | 18 19 | 37 | 2780 |
| 30 | VEGFA | 6p | 43 Mb | 0 | 43-0=43 | 24 26 | 50 | 3609 |
| 31 | IL7 | 8q | 78 Mb | 142 | 142-78=64 | 33 30 | 63 | 2016 |
| 32 | MMP1 | 11q | 102 Mb | 134 Mb | 134-102=32 | 29 29 | 58 | 1971 |
| 33 | MCP1 | 17q | 34 Mb | 83 | 83-34=49 | 29 31 | 60 | 741 |
| 34 | Flt3L | 19q | 49 Mb | 58 Mb | 58-49=9 | 18 20 | 38 | 1050 |
| 35 | CD8A | 2p | 86 Mb | 0 | 86-0=86 | 23 26 | 49 | 2106 |
| 36 | TNF | 6p | 31 Mb | 0 | 31-0=31 | 23 23 | 46 | 1678 |
| 37 | CCL28 | 5p | 43 Mb | 0 | 43-0=43 | 28 33 | 61 | 3126 |

***** multisystem inflammatory syndrome in children, MIS-C

**S2 Table. (continued)**

| Cytokines | gene ID | chr | gene locus | telomere locus | gene to telomere | A, T (%) | A + T (%) | FL (bp) |
| --- | --- | --- | --- | --- | --- | --- | --- | --- |
| 38 | IL10RB | 21q | 33 Mb | 46 Mb | 46-33=13 | 27 26 | 53 | 1941 |
| 39 | CD40 | 20q | 46 Mb | 64 Mb | 64-46=18 | 26 24 | 50 | 1682 |
| 40 | TWEAK | 17p | 7 Mb | 0 | 7-0=7 | 18 21 | 39 | 1377 |
| 41 | MMP10 | 11q | 102 Mb | 134 | 134-102=32 | 27 31 | 58 | 1759 |
| 42 | CCL25 | 19p | 8 Mb | 0 | 8-0=8 | 19 25 | 44 | 1002 |
| 43 | IL-10RA | 11q | 117 Mb | 135 | 135-117=18 | 22 23 | 45 | 3653 |
| 44 | NT-3 | 12p | 5 Mb | 0 | 5-0=5 | 27 27 | 54 | 1168 |
| 45 | TRAIL | 3q | 172 Mb | 198 | 198-172=26 | 33 28 | 61 | 1876 |
| 46 | FGF-23 | 12p | 4 Mb | 0 | 4-0=4 | 27 27 | 54 | 3002 |
| 47 | IL-15RA | 10p | 5 Mb | 0 | 5-0=5 | 23 22 | 45 | 1566 |
| 48 | uPA | 10q | 73 Mb | 133 | 133-73=60 | 23 25 | 48 | 2343 |
| 49 | ADA | 20q | 44 Mb | 64 | 64-44=20 | 24 19 | 43 | 1496 |
| 50 | TNFRSF9 | 1p | 7 Mb | 0 | 7-0=7 | 27 30 | 57 | 5872 |
| 51 | IL-18A | 11q | 112 Mb | 134 | 134-112=22 | 26 26 | 52 | 628 |
| 52 | CD5 | 11q | 61 Mb | 134 | 134-61=73 | 22 22 | 44 | 3127 |
| 53 | FGF-19 | 11q | 69 Mb | 134 | 134-69=65 | 20 25 | 45 | 1821 |
| 54 | IL-12B | 5q | 159 Mb | 181 | 181-159=22 | 29 28 | 57 | 2364 |
| 55 | CST5 | 20p | 23 Mb | 0 | 23-0=23 | 21 25 | 46 | 755 |
| 56 | TNFB | 6p | 31 Mb | 0 | 31-0=31 | 22 22 | 44 | 1416 |
| 57 | SIRT2 | 19q | 38 Mb | 58 | 58-38=20 | 22 20 | 42 | 1862 |
| 58 | STAMBP | 2p | 73 Mb | 0 | 73-0=73 | 26 31 | 57 | 6412 |
| 59 | 4E-BP1 | 8p | 38 Mb | 0 | 38-0=38 | 21 17 | 38 | 827 |
| 60 | CD244 | 1q | 160 Mb | 248 | 248-160=148 | 25 28 | 53 | 2495 |
| 61 | DNER | 2q | 229 Mb | 241 | 241-229=12 | 23 28 | 51 | 3257 |
| 62 | CCL11 | 17q | 34 Mb | 83 | 83-34=49 | 27 30 | 57 | 1005 |
| 63 | AXIN1 | 16q | 0 | 0 | 0-0-0 | 20 17 | 37 | 3707 |
| 64 | TRANCE | 13q | 42 Mb | 114 | 114-42=72 | 28 30 | 58 | 2201 |
| 65 | SCF | 12q | 88 Mb | 132 | 132-88=44 | 23 24 | 47 | 3168 |
| 66 | CD6 | 11q | 60 Mb | 134 | 134-60=74 | 19 20 | 39 | 3252 |
| 67 | CASP-8 | 2q | 201 Mb | 241 | 241-201=40 | 28 29 | 57 | 2914 |
| 68 | CXCL11 | 4q | 76 Mb | 189 | 189-76=113 | 34 32 | 66 | 1479 |
| 69 | MCP-4 | 17q | 34 Mb | 83 | 83-34=49 | 26 30 | 56 | 823 |
| 70 | CXCL1 | 4q | 73 Mb | 189 | 189-73=116 | 26 30 | 56 | 1174 |
| 71 | CXCL6 | 4q | 73 Mb | 189 | 189-73=116 | 27 35 | 62 | 1537 |
| 72 | ST1A1 | 16p | 28 Mb | 0 | 28-0=28 | 26 20 | 46 | 1254 |
| 73 | CXCL5 | 4q | 73 Mb | 189 | 189-73=116 | 28 36 | 64 | 2436 |

**References for this section**

1. Gruber CN, Patel RS, Trachtman R, Lepow L, Amanat F, Krammer F, et al. Mapping Systemic Inflammation and Antibody Responses in Multisystem Inflammatory Syndrome in Children (MIS-C). Cell. 2020;183(4):982-95 e14. Epub 2020/09/30. doi: 10.1016/j.cell.2020.09.034. PubMed PMID: 32991843; PubMed Central PMCID: PMCPMC7489877.
