# Supplementary material for: Mutability of druggable kinases and pro-inflammatory cytokines by their proximity to telomeres and A+T content
Source: PLoS One. 2023 Apr 27;18(4):e0283470. doi: 10.1371/journal.pone.0283470 (PMC10138820; doi:10.1371/journal.pone.0283470)
Supplement: S3 Table — * Not significant, NS; ** significant, Sig. (DOCX) [file pone.0283470.s003.docx]

Supporting Information

Mutability of druggable kinases and pro-inflammatory cytokines by their proximity to telomeres and A+T content

Ian McKnight^1^, Regan Raines^1^, Hunter White^1^,

Nasim Nosoudi^1^, Chan Lee^2^, Peter H.U. Lee^3,4^, Joon W. Shim^1^,*

Correspondence to: [shim@marshall.edu](mailto:shim@marshall.edu)

**This file includes:**

S3 Table

**S3 Table. Two factor characteristics of druggable kinases and select approved drugs.**

| Class of druggable proteins | **Druggable kinase candidates** | **Approved kinase drugs** | **Approved cytokine drugs** |
| --- | --- | --- | --- |
| **Less mutable** target (proportion);  = Factor-disease no match | 18 %  (23 of 129) | 33 %  (1 of 3) | 17 %  (1 of 6) |
| - Resulting top list | GK2  PIP5K1A  UCK2  PRKAG1  PHKA1 | EGFR  (Certain mutant forms of which are irreversibly targeted by osimertinib) | IL-23  (By guselkumab) |
| **More mutable** target (proportion);  = Factor-disease match | 82 %  (106 of 129) | 67 %  (2 of 3) | 83 %  (5 of 6) |
| - Resulting top list | CLK5  PRPF4B  PANK3  PDIK1L  PIK3C2G | PDGFR  (By imatinib) | TNF (by golimumab)  IL-17A (by secukinumab)  IL-6 (by siltuximab)  GDF8 (by Luspatercept)  VEGF (by bevacizumab/lucentis) |
| No. of genes | n=129 | n=3 | n=6 |
| Size-proximity correlation (Pearson) | **NS*** | ***-*** | **NS** |
| Size-A+T correlation (Pearson) | ***Sig.***** | **-** | **NS** |
| Relative size of genes (class-wise) | Longer | Longer | Shorter |

***** Not significant, NS; ** significant, Sig.
