# Supplementary material for: Mutability of druggable kinases and pro-inflammatory cytokines by their proximity to telomeres and A+T content
Source: PLoS One. 2023 Apr 27;18(4):e0283470. doi: 10.1371/journal.pone.0283470 (PMC10138820; doi:10.1371/journal.pone.0283470)
Supplement: S4 Table — * Copy number variation or de novo mutations found in mice after ionizing radiation2, CNV; ** A+T content of the entire chromosome calculated. (DOCX) [file pone.0283470.s004.docx]

Supporting Information

Mutability of druggable kinases and pro-inflammatory cytokines by their proximity to telomeres and A+T content

Ian McKnight^1^, Regan Raines^1^, Hunter White^1^,

Nasim Nosoudi^1^, Chan Lee^2^, Peter H.U. Lee^3,4^, Joon W. Shim^1^,*

Correspondence to: [shim@marshall.edu](mailto:shim@marshall.edu)

**This file includes:**

S4 Table

**S4 Table. Two factor characteristics of genetic loci in mice exposed to ionizing radiation.**

| CNV* loci[1] | CNV ID | chr | gene start | gene end | P or Q arm? | CNV to telomere | A + T ** (%) | FL (bp) |
| --- | --- | --- | --- | --- | --- | --- | --- | --- |
| 1 | CNVc1 | 11 | 25.9 M | 25.91 M | q arm | 95.10 | 56.00 | 4449 |
| 2 | CNVa8 | 4 | 21.33M | 21.34 | q arm | 134.70 | 55.40 | 15240 |
| 3 | CNVa1 | 1 | 7.8 M | 11.88 | q arm | 186.20 | 62.10 | 3989970 |
| 4 | CNVa13 | 5 | 65.68 M | 65.69 | q arm | 86.00 | 56.30 | 5687 |
| 5 | CNVa7 | 8 | 18.70 M | 18.76 | q arm | 110.24 | 60.10 | 58814 |
| 6 | CNVab12 | 19 | 57.18 M | 57.2 | q arm | 4.00 | 54.30 | 13637 |
| 7 | CNVa36 | 16 | 25.33 M | 25.37 | q arm | 71.50 | 50.80 | 40306 |
| 8 | CNVa15 | 5 | 78.79 M | 89.12 | q arm | 71.00 | 56.30 | 10334213 |
| 9 | CNVb35 | 13 | 76.29 M | 79.52 | q arm | 42.50 | 59.60 | 3235174 |
| 10 | CNVb54 | 12 | 100.82 M | 100.83 | q arm | 19.00 | 56.60 | 4453 |
| 11 | CNVb14 | 16 | 40.04 M | 40.04 | q arm | 58.00 | 50.80 | 4494 |
| 12 | CNVb16 | 11 | 41.63 M | 41.63 | q arm | 79.40 | 56.00 | 4514 |
| 13 | CNVb32 | 1 | 72.27 M | 72.282 | q arm | 121.73 | 62.10 | 9125 |
| 14 | CNVb6 | 14 | 15.30 M | 17.774 | q arm | 108.80 | 51.50 | 2468734 |
| 15 | CNVb28 | 15 | 61.77 M | 61.788 | q arm | 41.93 | 50.50 | 9491 |
| 16 | CNVb31 | 16 | 72.13M | 72.166 | q arm | 25.85 | 50.80 | 29387 |
| 17 | CNVb18 | 4 | 48.96M | 49.017 | q arm | 107.00 | 55.40 | 48214 |
| 18 | CNVb57 | 14 | 114.84M | 116.324 | q arm | 9.80 | 51.50 | 1482942 |
| 19 | CNVb15 | 16 | 41.62M | 41.695 | q arm | 57.00 | 50.80 | 66816 |
| 20 | CNVab12 | 19 | 57.18M | 57.202 | q arm | 3.90 | 54.30 | 13637 |

* copy number variation or de novo mutations found in mice after ionizing radiation[1], CNV; ** A+T content of the entire chromosome calculated

**References for this section**

1. Adewoye AB, Lindsay SJ, Dubrova YE, Hurles ME. The genome-wide effects of ionizing radiation on mutation induction in the mammalian germline. Nat Commun. 2015;6:6684. Epub 2015/03/27. doi: 10.1038/ncomms7684. PubMed PMID: 25809527; PubMed Central PMCID: PMCPMC4389250.
